# Percutaneous absorption of thirty-eight organic solvents *in vitro* using pig skin

**DOI:** 10.1371/journal.pone.0205458

**Published:** 2018-10-31

**Authors:** Linda Schenk, Matias Rauma, Martin N. Fransson, Gunnar Johanson

**Affiliations:** Unit of Work Environment Toxicology, Institute of Environmental Medicine, Karolinska Institutet, Stockholm, Sweden; Aristotle University of Thessaloniki, GREECE

## Abstract

Percutaneous absorption is highly variable between chemicals but also within chemicals depending on exposure conditions and experimental set up. We tested a larger number of organic solvents with the same experimental set up, using skin from new-born piglets and static diffusion cells. Thirty-six common organic solvents were studied neat (and 31 of them also in water dilution): acetone, acetonitrile, n-butanol 2-butanone 2-butoxyethanol, 1-butoxy-2-propanol, n-butyl acetate, butyl acrylate, cyclohexane, cyclohexanone, 1,2-dichloroethane, dichloromethane, ethanol, 2-ethoxyethanol, ethyl acetate, ethyl acrylate, ethylbenzene, furfuryl alcohol, n-hexane, 2-hexanone, 2-isopropoxyethanol, methanol, 1-methoxy-2-propanol, methyl acrylate, 3-methyl-1-butanol, methyl tertiary butyl ether, 4-metyl-2-pentanol, methyl methacrylate, 2-propanol, 2-propen-1-ol, 2-propoxyethanol, 1-propoxy-2-propanol, styrene, trichloromethane, toluene and m-xylene. In addition, a mixture of 2-methylbutyl acetate and n-pentyl acetate was tested. For most of the solvents, little or no percutaneous absorption data have been published. Lag times, steady-state fluxes and apparent permeability coefficients were obtained from the time courses of solvent appearance in the receptor medium, as measured by gas chromatography. The use of the same methodology and kind of skin resulted in small variability within experiments, underlining the need for consistent methodology for useful results for developing predictive models. Furthermore, a comparison of the neat and diluted data shows that water dilution affects all these variables and that the direction and magnitude of the effects vary between chemicals. This comparison strongly supports that prediction of percutaneous absorption of neat and water diluted chemicals requires different models.

## Introduction

Understanding percutaneous absorption of organic solvents is important in many areas, such as prediction of the time-to-effect of topically administered medications, the retention and behaviours of cosmetics on the skin, or risk assessment of unintended exposures. The demand for percutaneous absorption data for risk assessment purposes has increased with the introduction of the REACH legislation in the European Union, as manufacturers and producers of chemicals are required to evaluate the safe use of their products considering all relevant exposure routes, including dermal exposure.

For risk assessment of occupational exposures, solvents are of special importance due to extensive occupational use. Both inhalation and dermal exposure may contribute to systemic toxicity, meanwhile, successful reduction of ambient air levels increases the relative importance of dermal exposure. In the regulation of occupational exposure, the so-called “skin notation” is commonly used (in conjunction with occupational exposure limits, OELs) to identify chemicals that are easily taken up through the skin, and/or may cause or contribute to systemic toxicity. However, the criteria for skin notations, and how they are applied in practice, differ substantially between standard-setters [1–3]).

Among the difficulties to assign skin notation is the lack of relevant and reliable absorption data. A review of available studies for 108 different chemicals showed a huge variation in dermal permeability between as well as within chemicals [4]. This highlights the need for evaluating appropriateness of dermal absorption data. The kind of data most relevant for skin notations and other risk assessments of human skin exposure, i.e. human or *in vivo* dermal exposure toxicity, are rare due to ethical, practical and economical restrictions. Therefore, route-to-route extrapolation will be required for most chemicals. The same restrictions apply also to human and *in vivo* toxicokinetic data, underscoring the need for *in vitro* systems to evaluate percutaneous absorption and, in a longer perspective, to develop or improve computational predictive models.

The static diffusion cell, or Franz cell [5], is a commonly used tool to measure percutaneous absorption *in vitro*. The most common physical quantities used to describe the absorption process of a specific chemical can be derived from Fick’s laws of diffusion. Given a homogenous medium exposed to a chemical Fick’s first law can be used to calculate the steady-state flux *J*_*SS*_ and the permeability coefficient *K*_*p*_. These two physical quantities may then be used to develop predictive models, such as quantitative structure–activity relationship (QSAR) or quantitative structure-property relationship (QSPR) models [6,7]. Such models for percutaneous absorption have been the scope of a multitude of publications, e.g. [8–14], as well as evaluations of their performance [11, 15–18]. These models are mainly based on linear methods, which may not be sufficient for modelling the dynamics of percutaneous absorption. There are also few examples of different machine learning techniques [18–21], which show some promise. Deep learning methods in particular might be useful in future analysis of skin permeation data. Deep learning has been applied in a few cases of pharmaceutical and toxicological research [22] and performed well in the 2014 *Tox21 challenge* on prediction of nuclear receptor signaling and stress pathway assays [23].

A challenge in the development of linear as well as nonlinear approaches to predictive modelling of skin permeation is the limited availability of experimental data. Thus, the skin permeation models cited above are based on data for roughly 10 to 250 compounds whereas the Tox21 challenge participants had access to standardised data for 12 000 compounds [23]. Whichever predictive skin permeation model is chosen, the accuracy will depend on the input data and, as shown by e.g. Johanson and Rauma [4], these data are highly variable. For example, the donor species, location on the body and skin preparation all have a major impact on the results. Additionally, the experimental protocol, experimental and analytical equipment will influence study results [24,25]. For instance, experimental permeation data from diffusion cells using synthetic membranes, reducing the intra- and interspecies variability, may still comprise both inter- and intra-laboratory variability [26,27]. Comparisons of the static diffusion cells with flow-through diffusion cells, however, have shown that these two *in vitro* systems yield similar results [28–30].

The purpose of the present study was to evaluate the dermal penetration potential of a number of chemicals relevant for the occupational setting, namely organic solvents. Permeation studies of 38 common organic solvents were performed with skin from new-born piglets using the same experimental set up with static diffusion cells. Because the properties of neat chemicals and water dilutions may differ significantly, both these solutions were tested. Estimates of the lag time *t*_*lag*_, the steady-state flux *J*_*SS*_, and the apparent permeability coefficient *K*_*p*_ are presented.

## Materials and methods

### 2.1 Chemicals

Acetone (CAS 67-64-1, purity ≥99.5%), acetonitrile (75-05-8, ≥99.8%), n-butanol (71-36-3, 99.5%), trichloromethane (67-66-3, ≥99.0%), dichloromethane (75-09-2, ≥99.5%), ethyl acetate (141-78-6, ≥99.5%), 3-methyl-1-butanol (123-51-3, ≥99.0%), 2-butanone (78-93-3, ≥99.5%) and m-xylene (108-38-3, ≥99.0%) were obtained from Merck (Darmstadt, Germany).

2-Butoxyethanol (CAS 111-76-2, purity 99%), 2-ethoxyethanol (110-80-5, 98%) and toluene (108-88-3, ≥99.5%) were obtained from Kebo Lab (Stockholm, Sweden).

n-Butyl acetate (CAS 123-86-4, purity ≥99.0%), 1-butoxy-2-propanol (5131-66-8, ≥99.0%), butyl acrylate (141-32-2, ≥99.0%), cyclohexane (110-82-7, 99.9%), cyclohexanone (108-94-1, 99%), Furfuryl alcohol (98-00-0, 99%), 2-hexanone (591-78-6, 98%), 2-isopropoxyethanol (109-59-1, 99%), methanol (67-56-1, 99%), 1-methoxy-2-propanol (107-98-2, 98%), methyl acrylate (96-33-3, 99.0%), methyl tertiary butyl ether (1634-04-4, 99.8%), 4-metyl-2-pentanol (108-11-2, 98%), 2-propanol (67-63-0, ≥99.5%) and 1-propoxy-2-propanol (1569-01-3, 99%) were obtained from Sigma Aldrich (Steinheim, Germany). A commercially available mixture of n-pentyl acetate (628-63-7) and 2-methylbutyl acetate (624-41-9) was obtained from Sigma Aldrich (Steinheim, Germany) in a 65%/35% mixture having 99% purity, proportions were 60% and 40% according to our analysis by gas chromatography.

Ethanol (CAS 64-17-5, purity 99%) was obtained from Kemetyl (Stockholm, Sweden).

2-Propen-1-ol (CAS 107-18-6, purity ≥99.5%), 1,2-dichloroethane (107-06-2, ≥99.5%), ethyl acrylate (140-88-5, ≥99.0%), ethylbenzene (100-41-4, ≥98.0%), n-hexane (110-54-3, ≥98.0%), methyl methacrylate (80-62-6, ≥99.0%), 2-propoxyethanol (2807-30-9, ≥99.0%) and styrene (100-42-5, ≥99.0%) were obtained from Fluka (Buchs, Switzerland).

For the dilution studies, the three solvents with limited miscibility with water, m-xylene, cyclohexane and ethylbenzene, were diluted in degassed phosphate buffered saline (1000–3, Sigma–Aldrich, Steinheim, Germany) containing 6% PEG-20 oleyl ether (P5641, Sigma-Aldrich, Steinheim, Germany). Remaining chemicals were diluted in laboratory grade deionized water. All reported percentages are calculated by volume.

### 2.2 Skin

Piglets (Duroc) that had died of natural causes (at birth or first week of life) were obtained from local commercial breeders. As this source of skin is categorised as slaughter waste, it is exempt from the Swedish Board of Agriculture’s requirements on ethical vetting of research involving animals.

Pig skin has been shown to be similar to human skin with respect to *stratum corneum* and epidermal thickness as well as permeability [4,25,31,32]. Skin specifically from new-born pigs that died of natural causes has also been shown to be a suitable replacement for human skin by Cilurzo et al. [33], as their experimentally derived *in vitro* fluxes for seven benzoxaznones were within a factor of 2 from previously published data on human epidermis.

For early experiments we employed dermatomed skin (n = 10, labelled in Table 1), and then proceeded to use full-thickness skin. In both cases, skin pieces measuring approximately 8 x 5 cm^2^ were collected from the back and flank of the piglet. Each skin piece was stretched around the edges of a soft polyethene plate (21 x 3 x 0.7 cm^3^) and fastened to the sides of the plate with a staple gun. Another plate, wrapped in polyethene film, served as a lid and was placed on top of the skin and firmly and evenly fixed to the bottom plate by bolts and nuts. The mounted skin pieces were stored at -20°C. In those cases skin was dermatomed, it was taken out after 12 h and the frozen piece dermatomed (Model C, Padgett Instruments, Inc., Kansas City, MO). Dermatomed skin pieces were wrapped in aluminium foil and polyethene film, and then stored at -20°C until later use.

**Table 1:** Permeability data from static diffusion cell experiments using pig skin.

Compound CAS	# C	MW (g mol^-1^)	Log P	*c*_*donor*_ (%)	Skin	N	*t*_*lag*_ (min)	SE_*tlag*_ (min)	*J*_*ss*_ (g cm^-2^ h^-1^)	SE_*Jss*_ (g cm^-2^ h^-1^)	*K*_*p*_ (cm h^-1^)	SE_*Kp*_ (cm h^-1^)	*CV*_*Kp*_ (%)
*Alcohols*												** **	
Methanol	1	32.0	-0.63	100.0	Full	6	40.1	6.5	1.01E-02	7.55E-04	1.27E-02	9.55E-04	18
67-56-1				10.0	Split	6	23.4	1.6	7.03E-04	2.70E-04	8.89E-03	3.41E-03	94
Ethanol	2	46.1	-0.14	100.0	Split	6	22.4	2.5	5.71E-03	1.59E-03	7.23E-03	2.02E-03	68
64-17-5				10.0	Split	6	21.7	4.4	1.01E-03	4.46E-04	1.28E-02	5.65E-03	108
2-Propen-1-ol	3	58.1	0.21	100.0	Full	6	31.8	4.9	2.25E-03	1.08E-03	2.65E-03	1.27E-03	117
107-18-6				10.0	Full	6	36.3	5.1	3.76E-04	6.99E-05	4.43E-03	8.22E-04	46
2-Propanol	3	60.1	0.28	100.0	Split	5	18.0	2.1	5.96E-04	2.19E-04	7.59E-04	2.79E-04	82
67-63-0				10.0	Full	6	60.8	2.0	1.98E-04	2.22E-05	2.50E-03	2.81E-04	27
n-Butanol	4	74.1	0.84	100.0	Full	6	32.5	4.7	2.11E-04	3.23E-05	2.60E-04	3.99E-05	37
71-36-3				10.0	Full	6	68.5	8.5	4.43E-04	1.59E-04	5.46E-03	1.97E-03	88
3-Methyl-1-butanol	5	88.2	1.26	100.0	Full	6	28.7	5.5	2.57E-04	4.46E-05	3.16E-04	5.48E-05	43
123-51-3				1.0	Full	6	39.1	4.6	9.53E-05	3.81E-05	1.17E-02	4.69E-03	98
Furfuryl alcohol	5	98.1	0.45	100.0	Full	6	40.5	3.6	5.44E-04	9.46E-05	4.82E-04	8.37E-05	43
98-00-0				10.0	Full	6	64.2	9.4	5.09E-04	1.09E-04	4.50E-03	9.66E-04	52
4-Metyl-2-pentanol	6	102.2	1.68	100.0	Full	6	40.9	5.3	1.30E-04	2.89E-05	1.61E-04	3.58E-05	54
108-11-2				1.0	Full	6	68.5	8.4	3.29E-05	5.70E-06	4.08E-03	7.06E-04	42
*Chlorinated*													
Dichloromethane	1	84.9	1.34	100.0	Full	6	13.7	1.9	1.15E-02	8.51E-04	8.66E-03	6.42E-04	18
75-09-2				1.0	Full	6	22.4	3.5	4.18E-04	2.04E-04	3.15E-02	1.54E-02	120
Trichloromethane	1	119.4	1.52	100.0	Full	6	38.7	5.9	7.67E-03	7.66E-04	5.18E-03	5.18E-04	24
67-66-3				0.5	Full	6	28.4	5.7	4.96E-04	7.50E-05	6.70E-02	1.01E-02	37
1,2-Dichloroethane	2	99.0	1.83	100.0	Full	6	30.7	2.7	1.36E-03	1.79E-04	1.09E-03	1.43E-04	32
107-06-2				0.8	Full	6	22.0	5.4	5.96E-04	5.95E-05	5.94E-02	5.94E-03	24
*Aromatic*													
Toluene	7	92.1	2.54	100.0	Split	6	27.4	1.8	3.80E-04	2.78E-05	4.36E-04	3.20E-05	18
108-88-3						0							
Styrene	8	104.2	2.89	100.0	Full	6	90.3	14.6	7.11E-05	1.33E-05	7.86E-05	1.47E-05	46
100-42-5						0							
Ethylbenzene	8	106.2	3.03	100.0	Full	6	148.5	8.2	1.24E-04	1.39E-05	1.43E-04	1.60E-05	27
100-41-4						0							
m-Xylene	8	106.2	3.09	100.0	Split	6	69.0	1.8	6.27E-05	4.90E-06	7.29E-05	5.69E-06	19
108-38-3				1.0	Split	5	73.3	6.1	1.84E-05	3.10E-06	2.13E-03	3.60E-04	38
*Esters*													
Methyl acrylate	4	86.1	0.73	100.0	Full	6	11.9	2.3	1.01E-03	4.49E-05	1.06E-03	4.70E-05	11
96-33-3				1.0	Full	6	16.2	3.6	2.83E-04	2.74E-05	2.97E-02	2.86E-03	24
Ethyl acetate	4	88.1	0.86	100.0	Full	5	22.6	4.8	2.70E-03	6.48E-04	3.02E-03	7.25E-04	54
141-78-6				1.0	Full	6	19.1	4.4	8.84E-04	8.16E-05	9.88E-02	9.12E-03	23
Ethyl acrylate	5	100.1	1.22	100.0	Full	6	26.6	3.2	9.58E-04	2.35E-04	1.04E-03	2.54E-04	60
140-88-5				1.0	Full	6	23.1	2.1	3.97E-04	5.04E-05	4.29E-02	5.46E-03	31
Methyl methacrylate	5	100.1	1.28	100.0	Full	6	20.7	3.8	8.00E-04	8.90E-05	8.52E-04	9.47E-05	27
80-62-6				1.0	Full	6	19.1	3.5	2.89E-04	2.00E-05	3.07E-02	2.13E-03	17
n-Butyl acetate	6	116.2	1.85	100.0	Split	6	24.3	4.1	4.75E-04	4.49E-05	5.39E-04	5.10E-05	23
123-86-4				0.1	Full	6	34.1	7.7	3.33E-05	2.43E-06	3.79E-02	2.76E-03	18
Butyl acrylate	7	128.2	2.20	100.0	Full	6	56.6	5.0	1.78E-04	1.57E-05	1.98E-04	1.74E-05	22
141-32-2				0.1	Full	6	39.8	5.5	1.26E-05	1.35E-06	1.40E-02	1.50E-03	26
2-Methylbutyl acetate^a^	7	130.2	2.26	40.0	Full	6	59.0	3.9	2.23E-04	3.61E-05	6.37E-04	1.03E-04	40
624-41-9						0							
n-Pentyl acetate^a^	7	130.2	2.34	60.0	Full	6	54.7	5.4	6.26E-04	8.21E-05	1.19E-03	1.56E-04	32
628-63-7						0							
*Glycol ethers*													
2-Ethoxyethanol	4	90.1	-0.42	100.0	Full	6	58.5	2.5	9.19E-04	1.74E-04	9.88E-04	1.87E-04	46
110-80-5				10.0	Full	5	47.0	7.3	1.22E-04	1.76E-05	1.31E-03	1.90E-04	32
1-Methoxy-2-propanol	4	90.1	-0.49	100.0	Full	6	45.6	5.5	7.48E-04	1.33E-04	8.15E-04	1.45E-04	44
107-98-2				10.0	Full	6	63.0	6.1	9.60E-05	1.64E-05	1.05E-03	1.78E-04	42
2-Isopropoxyethanol	5	104.1	0.00	100.0	Full	6	16.7	2.8	1.23E-04	1.92E-05	1.36E-04	2.12E-05	38
109-59-1				10.0	Full	6	51.3	3.5	7.69E-05	1.38E-05	8.51E-04	1.53E-04	44
2-Propoxyethanol	5	104.1	0.08	100.0	Full	6	46.1	5.8	1.89E-04	1.89E-05	2.08E-04	2.07E-05	24
2807-30-9				10.0	Full	6	60.7	6.4	1.77E-04	2.89E-05	1.95E-03	3.17E-04	40
2-Butoxyethanol	6	118.2	0.57	100.0	Full	6	66.0	9.2	7.78E-05	1.50E-05	8.64E-05	1.67E-05	47
111-76-2				10.0	Full	6	66.3	5.9	7.93E-04	1.52E-04	8.81E-03	1.69E-03	47
1-Propoxy-2-propanol	6	118.2	0.49	100.0	Full	4	23.3	10.8	2.03E-04	4.34E-05	2.30E-04	4.90E-05	43
1569-01-3				10.0	Full	6	50.4	4.0	3.30E-04	6.21E-05	3.73E-03	7.02E-04	46
1-Butoxy-2-propanol	7	132.2	0.98	100.0	Full	6	33.6	5.1	1.44E-04	3.37E-05	1.64E-04	3.83E-05	57
5131-66-8				7.0	Full	6	40.6	2.8	5.22E-04	4.66E-05	8.48E-03	7.57E-04	22
*Ketones*													
Acetone	3	58.1	-0.24	100.0	Full	6	13.1	3.2	1.96E-03	4.47E-04	2.49E-03	5.69E-04	56
67-64-1				10.0	Full	6	37.9	7.5	1.01E-03	2.13E-04	1.29E-02	2.71E-03	52
2-Butanone	4	72.1	0.26	100.0	Full	6	23.6	3.1	1.41E-03	1.65E-04	1.75E-03	2.05E-04	29
78-93-3				10.0	Full	6	36.3	4.7	8.67E-04	1.43E-04	1.08E-02	1.77E-03	40
Cyclohexanone	6	98.1	1.13	100.0	Split	6	15.4	3.2	1.81E-03	5.43E-04	1.91E-03	5.72E-04	73
108-94-1						0							
2-Hexanone	6	100.2	1.24	100.0	Full	6	26.2	3.6	5.95E-04	1.16E-04	7.33E-04	1.43E-04	48
591-78-6				1.0	Full	6	28.8	4.1	3.13E-04	3.50E-05	3.85E-02	4.31E-03	27
*Miscellaneous*													
Acetonitrile	2	41.1	-0.15	100.0	Full	6	17.9	2.6	5.85E-04	1.68E-04	7.45E-04	2.13E-04	70
75-05-8				10.0	Full	6	35.5	3.2	8.47E-04	2.05E-04	1.08E-02	2.61E-03	59
Methyl tertiary butyl ether	5	88.2	1.34	100.0	Full	6	22.2	3.4	1.94E-03	4.33E-04	2.63E-03	5.85E-04	55
1634-04-4				1.0	Full	6	31.2	5.7	8.93E-05	1.36E-05	1.21E-02	1.83E-03	37
Cyclohexane	6	84.2	3.18	100.0	Full	6	165.4	14.7	6.09E-05	1.29E-05	7.91E-05	1.67E-05	52
110-82-7				0.1	Full	4	205.1	36.2	6.72E-06	1.38E-06	8.72E-03	1.79E-03	41
n-Hexane	6	86.2	3.29	100.0	Split	6	63.1	5.0	9.15E-04	2.10E-04	1.41E-03	3.23E-04	56
110-54-3						0							

CAS, Chemical Abstracts Service Registry Number; # C, number of carbon atoms; CV, Coefficient of Variation (same for *J*_*SS*_ and *K*_*p*_); MW, molecular weight; *c*_*donor*_, volume concentration of chemical in donor compartment; N, number of experiments; *t*_*lag*_, lag time; *J*_*SS*_, flux at steady-state; *K*_*p*_, permeability coefficient; Log P, logarithm of the octanol:water partition coefficient (estimated using US EPA[73]); *SE*, standard error of the mean. Split, split-thickness skin; Full, full-thickness skin.

^a^2-Methylbutyl acetate (40%) and n-pentyl acetate (60%) were tested as a mixture.

Twenty four hours before study the skin pieces were thawed for 15 minutes in room temperature. Thereafter the thickness was measured by using a micrometer (293-661-10, Mitutoyo) and integrity was checked by an ohm meter (Fluke 111, Fluke Corporation, Everett, WA, USA). Pieces with a resistance below 50 kΩ were discarded. This cut-off was validated against in house measurements of intact and damaged skin pieces. The skin pieces were stored overnight in saline at +8°C prior to permeation measurements.

### 2.3 Franz cell studies

Six jacketed static Franz cells (orifice diameter 9 mm, corresponding to a skin exposure area of 0.64 cm^2^, receptor volume 5.0–5.4 mL, model number 4G-01-00-090-05, Permegear, Bethlehem, PA, USA) were mounted in a magnetic stirrer (HP 6 Variomag, H+P Labortechnik, Munich, Germany) and kept at 32°C [34] by means of circulating water from a thermostatted water bath (21 AT, Heto, Allreød, Denmark).

Degassed phosphate buffered saline (1000–3, Sigma–Aldrich, Steinheim, Germany) containing 6% PEG-20 oleyl ether (P5641, Sigma-Aldrich, Steinheim, Germany), according to OECD guidelines [34], was used as receptor fluid. The receptor compartment was kept well stirred using Teflon coated magnets. Skin pieces were mounted onto the Franz cells one hour before start of exposure.

At start of experiment, the donor compartment was filled with excess test chemical (approximately 1 ml, neat or diluted in water) and capped with a glass stopper. Experiments ran for 4 to 9 hours.

Aliquots of receptor fluid (50 μl) were sampled at predefined times (every 10 min first hour, every 20 min second hour, then every 30 min) using a gas-tight syringe (004250, SGE, Victoria, Australia). Samples were directly transferred to head-space glass vials, which were immediately capped and stored at +8°C for later analysis (within two days) by head-space gas chromatography.

### 2.4 Gas chromatographic analyses

The analyses were performed with a 6890+ GC (Hewlett Packard, Palo Alto, CA, USA), an 8700 GC (Perkin Elmer, Waltham, MA, USA) or a Clarus 500 (Perkin Elmer, Waltham, MA, USA) gas chromatograph equipped with a 10-m or 25-m Poraplot Q column and flame-ionization detector.

All studied chemicals were readily detected in the gas chromatographic analyses of receptor medium with limits of detection ranging from 0.1 μg/ml to 9.5 μg/ml (median 0.4 μg/ml) depending on chemical. To allow for quantitative analyses, standard curves were established for each test chemical. At least one concentration in each of the standard curves was well above the highest concentration achieved in the receptor fluid during the experiments. All standard curves were linear, showing that the maximum solubility of the receptor fluid was not exceeded for any of the tested chemicals.

### 2.5 Calculations

The steady-state flux, *J*_*SS*_ (g/cm^2^/h), was calculated from the exposed area of the skin (i.e. 0.64 cm^2^) and the slope of the steady-state region of the amount in the receptor medium versus time curve. From the steady-state flux the apparent permeability coefficient *K*_*p*_ was obtained as:

$$K_{p}=\frac{J_{SS}}{c_{donor}}$$ (1)

where *c*_*donor*_ is the concentration (g/cm^3^) of the chemical in the donor compartment exposing the skin. The lag time, *t*_*lag*_ (min), is defined as the time-point where the extrapolated steady-state region intersects with the x-axis.

Data management and calculations were performed in Microsoft Excel (2010). To avoid data points from the pre-steady state part of the curve, data points with sampling times below the estimated value of *t*_*lag*_ were excluded. Furthermore, for some chemical the time-concentration curve appeared to flatten out and these “post-steady state” values were also omitted.

Correlations between experimentally derived permeability measures and physicochemical characteristics were investigated using regression analyses in the software R (version 3.3.2). As pentyl acetate and 2-methylbutyl acetate were tested as a mixture, they were not included in these analyses.

## Results and discussion

The experimentally derived mean values of *t*_*lag*_, *J*_*SS*_, and *K*_*p*_ for each chemical and concentration are presented in Table 1. As expected, the tested solvents showed skin permeabilities (K_p_) ranging from “moderate” (10^−4^ cm/h) to “very high” (10^−2^ cm/h) according to previously proposed classification schemes [35,36].

**Fig 1:**
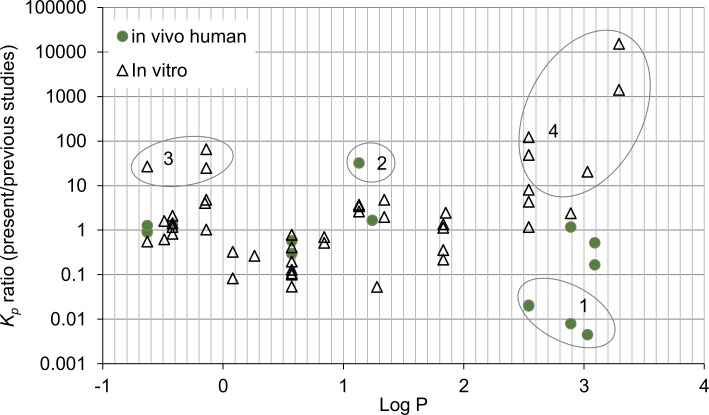
Ratios between apparent permeability coefficients (*K*_*p*_) for neat solvent obtained in the present study and those previously reported plotted over the log octanol:Water partition coefficient (log P estimated using US EPA [73]). Data were available for 20 chemicals, of which 9 had been tested on humans *in vivo* (13 ratios) and 18 on animal or human skin *in vitro* (47 ratios). Notes: 1: toluene, styrene and ethylbenzene [37–39]; 2: cyclohexanone [46]; 3: ethanol and methanol [55,62]; 4: ethylbenzene, n-hexane, toluene [52, 54].

Comparable percutaneous absorption data using infinite dose of, or occluded exposure to, neat chemical have been published previously for 20 of the 38 chemicals investigated herein. One aim of *in vitro* permeation experiments is to predict percutaneous permeation for humans. Human *in vivo* data in the neat was available for nine substances [37–49]. We also wished to compare our data to other studies using similar experimental conditions, i.e. *in vitro* using human or animal skin [50–72]. The ratio between *K*_*p*_ values of the present study and *K*_*p*_ values from previous studies are plotted against log octanol:water partition coefficient (log P) in Fig 1. Values of log P were estimated using the EPI Suite software [73].

For nine of the 13 available human *in vivo* studies the *K*_*p*_ values were similar to those obtained in the present study (Fig 1, circles). Dutkiewicz and Tyras [37–39] reported very high and seemingly unrealistic fluxes and *K*_*p*_ values for toluene, styrene and ethyl (note 1 in Fig 1). As discussed above for ethylbenzene, the calculations were based on amount left on the skin after exposure. This method may easily overestimate the absorption. Mraz and colleagues [46] estimated the percutaneous absorption of cyclohexanone by quantification of its metabolite 1,2-cyclohexanediol in urine collected up to 72 h after exposure (note 2). The remaining human *in vivo* studies, covering six chemicals, are in reasonable agreement (within one order of magnitude) with our present study.

Estimates of *K*_*p*_ from previous *in vitro* studies on neat chemicals are available for 18 chemicals and are in most cases (37 out of 47 comparisons covering 15 chemicals) within one order of magnitude of the *K*_*p*_ for neat chemicals derived in the present study (Fig 1). These studies cover skin from several species (human, hairless rat/rat, guinea pig, pig/minipig), varying thickness and both static and flow-through diffusion cells. There are no discernible trends concerning species and agreement with our data. Although rat skin is expected to yield higher permeability coefficients than human skin or pig skin, this seems not to be the case in most studies. The deviating results for ethanol and methanol (note 3) may be due to evaporation at the sampling stage [55] or during the exposures as Pendlington and colleagues [62] only recovered 40% of applied ethanol, although care was taken to avoid evaporation. The largest deviations were seen for the more hydrophobic chemicals (note 4; ethylbenzene, n-hexane, toluene). For n-hexane the ratios reached as high as 1400 [54] and 15000 [52]. The latter study employed physiological saline as receptor medium, which would reduce diffusion as compare to our receptor solution, however, it is unclear whether these factors would reduce diffusion this much.

The experimental variability is comparably low across our performed experiments. For 71 cases listed in Table 1, the coefficient of variation ranged from 11% to 120%. This maximal factor of two is substantially slower than the many orders of magnitude seen in between studies for many substances [4]. Furthermore, the coefficient of variation was in most cases below 50% (49 out of 71) and only above 75% in 7 cases. The higher coefficients of variation were primarily found among alcohols (n = 6). Although we cannot offer any explanation for this pattern, we note that the cases cover both water dilutions and neat solvents as well as both full- and split-thickness skin.

**Fig 2:**
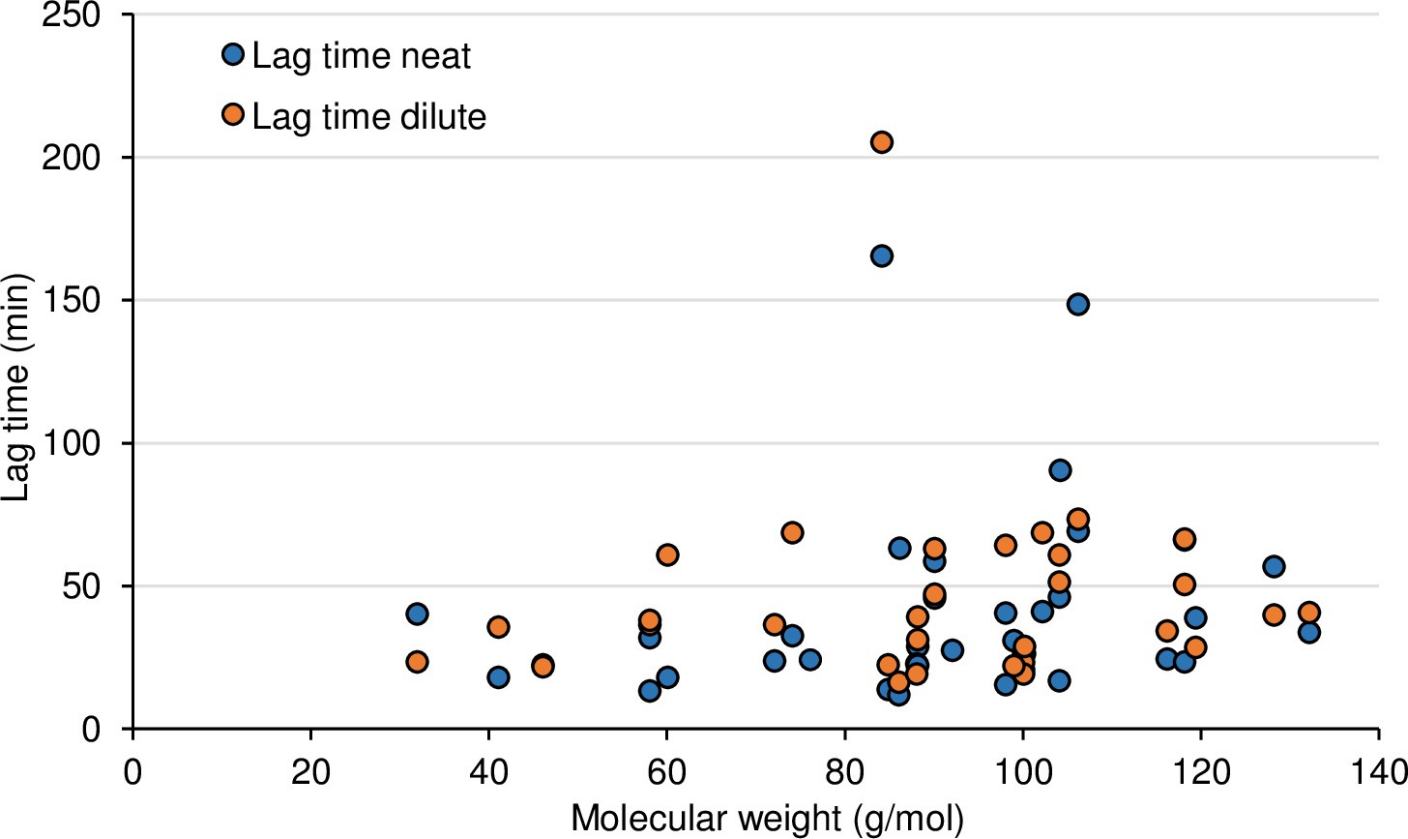
Lag time (min) plotted over molecular weight (g/mol).

In Fig 2, two substances attract attention due to long lag times, namely cyclohexane (165.5 min in neat and 205.1 min in 0.1% water dilution) and ethylbenzene (148.5 min, only tested neat). For cyclohexane experiments ran for 540 min (9h) and for ethylbenzene 360 min (6h). The long lag times decreases the confidence in the calculations of *J*_*SS*_ and *K*_*p*_ as steady state may not have been reached, in particular for ethyl benzene. For cyclohexane we have not found any other dermal permeation data. We found three studies presenting *J*_*SS*_ values for neat ethylbenzene. Dutkiewicz and Tyras [37] performed 10–15 min occluded exposures of volunteers to ethylbenzene *in vivo* and obtained a *J*_*SS*_ of 2.8∙10^−2^ g/cm^2^/h, i.e. 200 times higher than our value. In their study, the absorbed amount was defined as the difference between applied amount and amount remaining on the skin after exposure. This approach assumes that all chemical absorbed into skin is systemically absorbed, even though some may have evaporated after the occluded exposure ended. Tsuruta [52] used rat skin *in vitro* and physiological saline as the receptor medium. The resulting *J*_*SS*_ was 6.3∙10^−6^ g/cm^2^/h, i.e. 20 times lower than our value. As ethylbenzene is hydrophobic, the use of physiological saline as receptor medium may have led to an underestimation of *J*_*SS*_. Furthermore, the experiments ran for 3–6 hours and the *t*_*lag*_ was 2 hours, hence as in our experiments, steady state may not have been reached. The third and most recent study with ethylbenzene is that of Susten et al. [74] who exposed hairless mice *in vivo* to ethylbenzene. We consider this study to be the most reliable of the three, and it reported a *J*_*SS*_ of 2.2∙10^−3^ g/cm^2^/h, which is six-fold lower than our value.

Previous research indicates that molecular size is related to *t*_*lag*_, for instance Nielsen et al. [16] found a general trend of increasing *t*_*lag*_ with increased molecular weight in permeation experiments with 9 chemicals in aqueous solutions (MW 122 to 376.7 g/mol. Such a trend is obvious for the esters, where the lag time clearly increases with increasing MW (neat: intercept = -54.1, slope = 0.79, r^2^ = 0.71 p = 0.04; dilute: intercept = -32.9, slope = 0.56, r^2^ = 0.94, p = 0.001). However, for the overall material there was no strong correlation between molecular weight (neat: intercept = 14.5, slope = 0.29, r^2^ = 0.04 p = 0.2; dilute: intercept = 37.2, slope = 0.1, r^2^ = 0.005, p = 0.7). For number of carbons the correlations were statistically significant, but relatively small (neat: intercept = 3.4, slope = 7.9, r^2^ = 0.22 p = 0.004; dilute: intercept = 16.5, slope = 6.8, r^2^ = 0.14, p = 0.04). Thus, although some of the variability in lag time can be attributed to molecular size, other factors, among them polarity and dilution, seem to have a significant influence. For chemicals where both neat and diluted solutions were tested (n = 31) the neat solutions had shorter time lags than the corresponding diluted mixtures in two thirds of the cases. Methanol stands out as the only alcohol with a clearly longer time lag for the neat chemical (Table 1). Although we assume *stratum corneum* to constitute the main barrier, we cannot exclude that the difference in skin thickness between experiments for neat and diluted methanol played a role.

**Fig 3:**
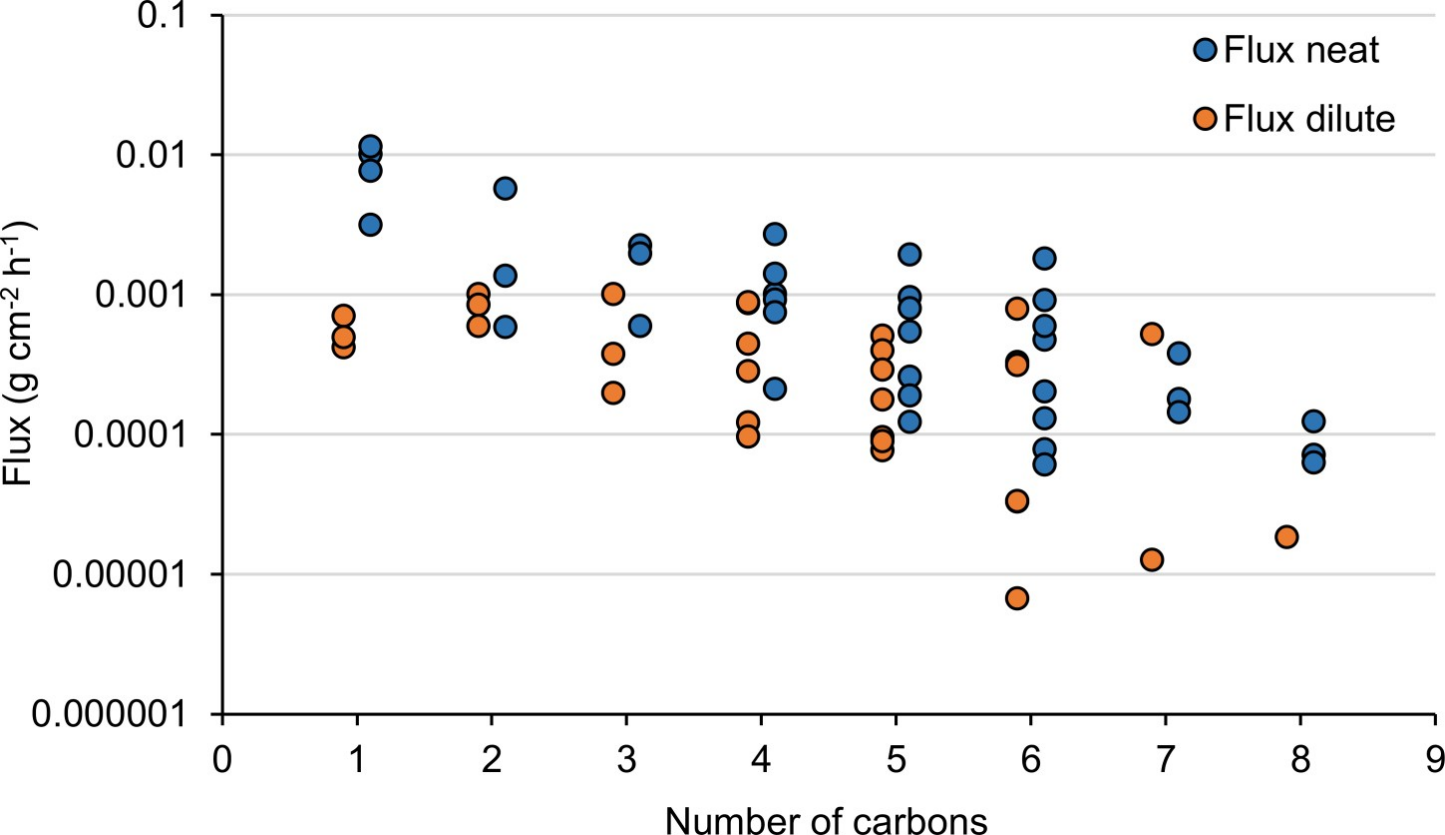
Steady state flux (mg cm^-2^ h^-1^) plotted in log scale over number of carbon atoms in the molecule.

The average steady-state fluxes (*J*_*SS*_) for each chemical and concentration are presented in Table 1. For neat substances, decreased flux with increasing number of carbon atoms is evident for most solvents (Fig 3). This relationship was statistically significant for both neat and diluted substances (neat: intercept = -2.0, slope = -0.25, r^2^ = 0.63, p<0.0001; dilute: intercept = -2.8; slope = -0.19; r^2^ = 0.35 p = 0.0004; log *J*_*SS*_). Diluted mixtures have in general a lower flux than neat chemicals, often only one tenth of the flux for the neat compound. As reported previously [49,69,75], 2-butoxyethanol is a striking exception with an opposite effect of dilution (Table 1). Bunge et al. identified that *J*_*SS*_ of 2-butoxyethanol was proportional to thermodynamic activity up to a concentration of 80% (by weight), at higher concentrations the decreased flux is likely due to dehydration of the skin [71].

**Fig 4:**
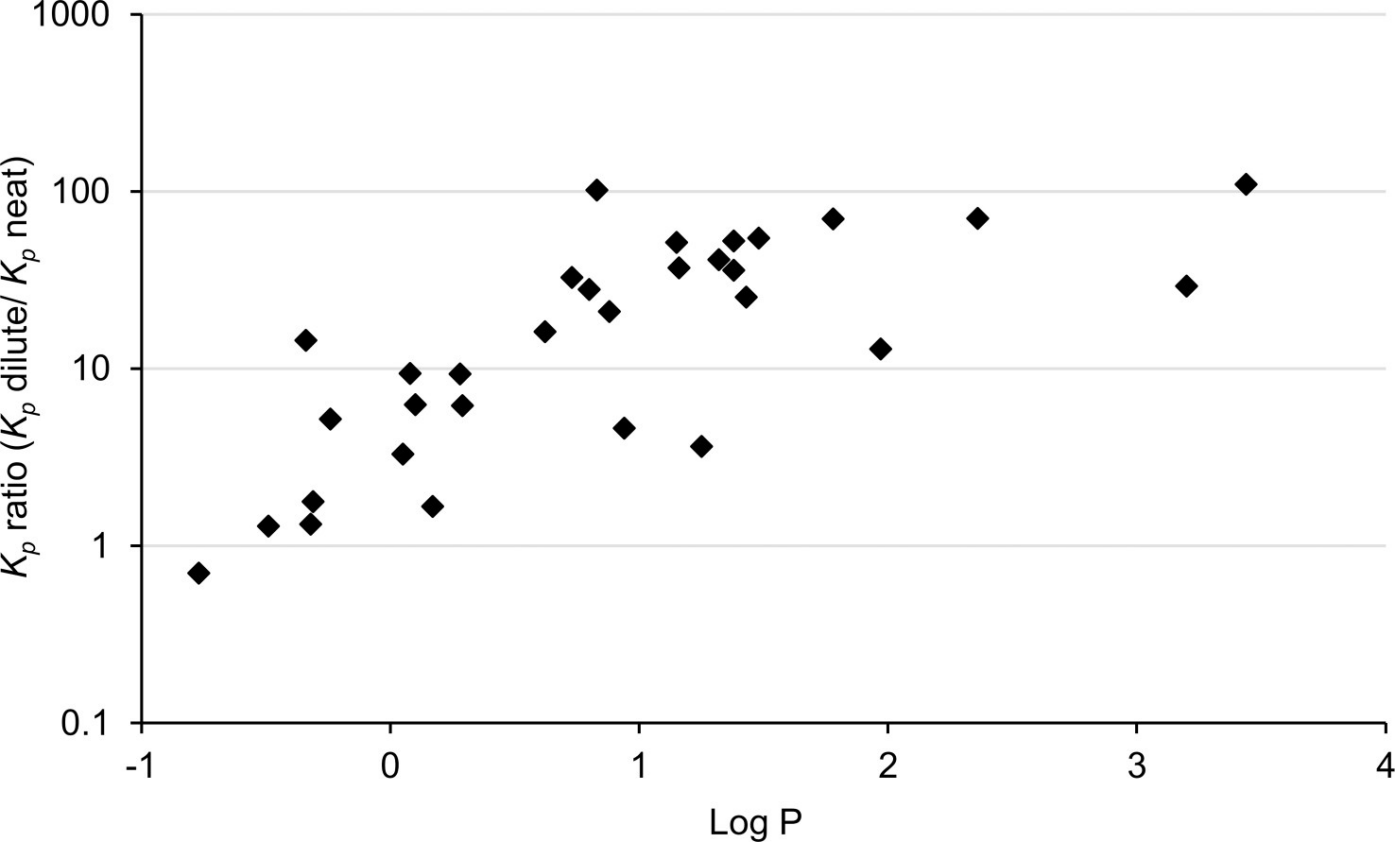
Ratio of apparent permeability coefficient (dilute / neat, log scale) plotted over the log octanol: Water partition coefficient (log P, estimated using US EPA[73]).

Of the neat chemicals, methanol had the highest apparent permeability coefficient (*K*_*p*_) of 1.3∙10^−2^ cm/h, while m-xylene had the lowest of 7.3∙10^−5^ cm/h (Table 1). The *K*_*p*_ values presented here are considered “apparent” as they are calculated using nominal concentrations and not thermodynamic activity. Because concentration and not activity was used in the denominator, we expect these apparent *K*_*p*_ values to be higher for diluted than for neat solvent, which also was the case for all but methanol (Table 1, Fig 4). However, as seen in Fig 4, the logarithm of the *K*_*p*_ ratio (dilute/neat) seems to increase with an increasing log P up to a log P of about 1.5. In particular for the amphiphilic solvents in our selection, the water dilution *vs* neat discrepancies in *K*_*p*_ may be partly explained by the substance altering the skin barrier function. This has been proposed for 2-buthoxyethanol by Bunge and colleagues [71] as well as for 2-hydroxypropyl acrylate by Frasch and colleagues [76]. For improved understanding of how water affects the *K*_*p*_, experimental data are needed on thermodynamic activity *vs* concentration for the test substances.

**Fig 5:**
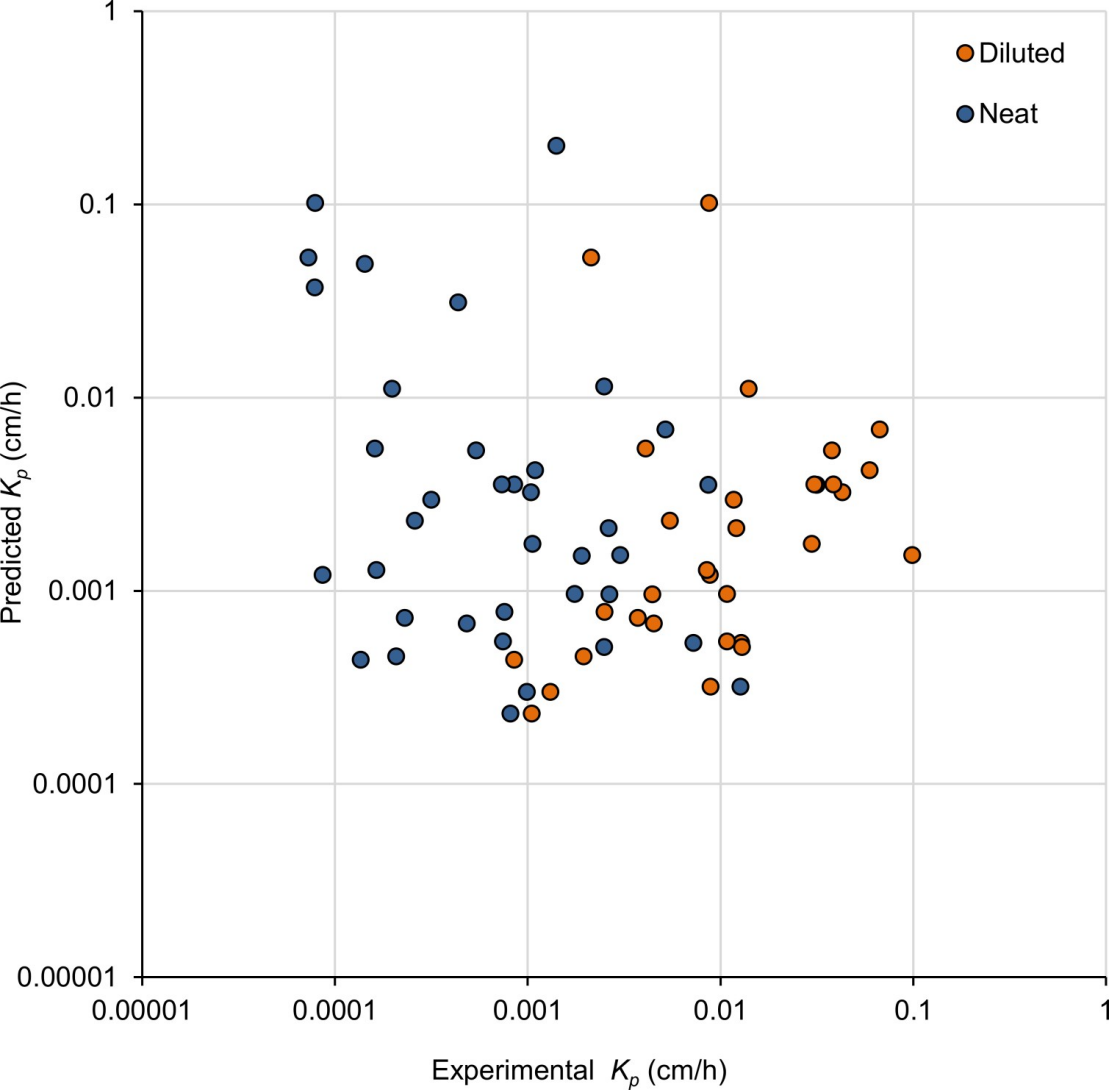
Predicted (EPISuite, US EPA [73]) versus experimental (present study) permeability coefficients (*K*_*p*_, cm/h) for neat (n = 36) and water diluted organic solvents (n = 31). Note the log scale on both axes, S1 Fig shows the data on linear scale.

The discrepancies in *K*_*p*_ between neat and diluted solvent also hold implications for predictive models. The present study allows comparison between predictions and experimental data for both neat and water dilution for 31 substances, studied using the same experimental set up. In Fig 5 we plot the EPISuite (US EPA [73]; similar to Potts and Guy[9]) predictions for the chemicals tested in neat and in water dilution over the experimentally derived values from the present study, log scale on both axes. The fit is poor (on a linear scale) for both neat (intercept = 0.02, slope = -1.9, r^2^ = 0.018, p = 0.4) and water diluted solvents (intercept = 0.009, slope = -0.08, r^2^ = 0.009, p = 0.6). Furthermore, the correlation has a negative slope, i.e. the lower the predicted *K*_*p*_, the higher the experimental *K*_*p*_ (see also S1 Fig). A previous study compared experimental permeation data for eleven substances in neat with outcomes for three different predictive models, finding that the model outcomes correlated well with each other but less so with experimental data [17]. Fig 5, and the differences in regression lines, furthermore illustrates that prediction of percutaneous absorption of neat and water diluted chemical requires markedly different models.

## Conclusions

We have measured lag times, steady-state fluxes, and apparent permeability coefficients for 38 organic solvents. For a number of them, this study contributes with the first, or one of few, experimental data sets on skin permeability. The present study adds to the body of evidence showing that *K*_*p*_ is concentration dependent, and that the influence of water dilution varies significantly between chemicals. Hence, dilution is a factor that needs to be considered in risk assessment of dermal exposures as well as incorporated in QSAR and other modelling efforts.

The variability between experiments was minimized as we used the same kind of skin and methodology, with minor variations, throughout. This is an important aspect, as the permeability for the same chemical has been found to vary by up to six orders of magnitude between different studies [4]. In the present study the coefficient of variation for our experiments ranged from 11% to 120% with a median of 45%. Only seven experiments yielded a coefficient of variation above 75%. Hence, overall, our experiments display low variability compared to that seen between studies [4]. The lower variability underlines the importance of a standardized and consistent methodology to achieve useful results e.g. for QSAR analyses. This aspect is probably far more important than the choice of human over pig skin. These two species are rather similar regarding *stratum corneum* and epidermal thickness as well as permeability [4,31].

Furthermore, human skin is in itself a source of inter- and intra-individual variability [25, 77]. Human skin may be difficult to obtain and, when obtained, it may be not be possible to control how it was sampled, from whom (donor age) and from where (body site). This is even more problematic if a large number of chemicals are to be covered. In conclusion, although human skin may seem preferable because of the intended application domain (i.e. percutaneous absorption and subsequent health risks to humans), well performed *in vitro* experiments with pig skin appear to be a better alternative.

## Supporting information

S1 Fig Predicted (EPISuite, US EPA [73]) versus experimental (present study) permeability coefficients (Kp, cm/h) plotted on a linear scale. (PDF)
