# Supplementary material for: Percutaneous absorption of thirty-eight organic solvents in vitro using pig skin
Source: PLoS One. 2018 Oct 31;13(10):e0205458. doi: 10.1371/journal.pone.0205458 (PMC6209206; doi:10.1371/journal.pone.0205458)
Supplement: S1 Fig — (PDF) [file pone.0205458.s001.pdf]

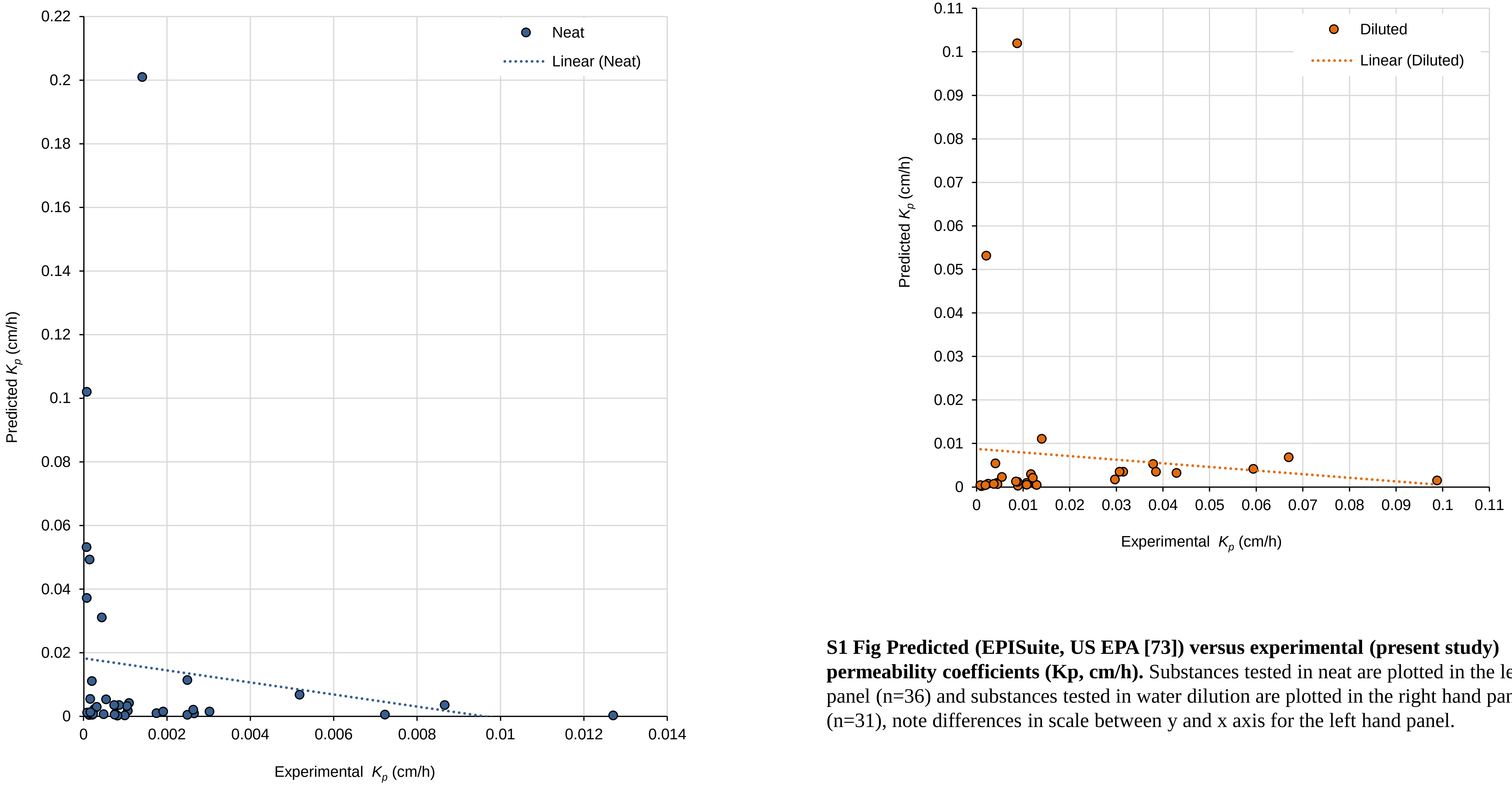

S1 Fig Predicted (EPISuite, US EPA [73]) versus experimental (present study) permeability coefficients (Kp, cm/h). Substances tested in neat are plotted in the left hand panel (n=36) and substances tested in water dilution are plotted in the right hand panel (n=31), note differences in scale between y and x axis for the left hand panel.
